# High-Performance Self-Powered Photodetector Based on Silver Triangular Nanoplate-Modified P3HT/ZnO Heterojunctions

**DOI:** 10.3390/s26092725

**Published:** 2026-04-28

**Authors:** Jun Zhou, Qian Qiao, Sijie Chen, Xuan Yu, Xiaoming Yu, Cao Li, Jian Zheng, Cunxi Zhang, Rui Wang

**Affiliations:** 1 School of Marine Engineering Equipment, Zhejiang Ocean University, Zhoushan 316022, China; 2 School of Information Engineering, Zhejiang Ocean University, Zhoushan 316022, China; 3 School of Naval Architecture and Maritime, Zhejiang Ocean University, Zhoushan 316022, China

**Keywords:** photodetector, ZnO, silver triangular nanoplate, P3HT

## Abstract

**Highlights:**

- Silver triangular nanoplates were introduced at the P3HT/ZnO heterojunction to construct a novel self-powered P3HT/AgTNPs/ZnO heterojunction photodetectors.
- The optimal responsivity enhancement ratio values occurs under irradiation of light at a wavelength of 525 nm. The responsivity values of the P3HT/AgTNPs-1/ZnO and P3HT/AgTNPs-2/ZnO devices increased by 3.24 and 4.21 times, respectively, compared with the reference P3HT/ZnO device.

**What is the main finding?**

- Silver triangular nanoplates were embedded at the P3HT/ZnO interface to form a composite structure. The photoelectric conversion efficiency of the plasmonic P3HT/AgTNPs/ZnO photodetectors significantly improved after by adding AgTNP nanostructures into the device in both the ultraviolet and visible wavelength regions.

**What are the implications of the main findings?**

- Confirm that embedding plasmonic nanoparticles with specific geometries (triangular plates) is an effective strategy to significantly enhance the performance of organic–inorganic heterojunction photodetectors.
- Provide a practical approach for boosting the sensitivity of self-powered devices, supporting the development of more efficient optical sensors for low-power IoT applications.

**Abstract:**

Self-powered photodetectors have attracted widespread attention in Internet of Things applications due to their low power consumption and high sensitivity. In this study, plasmonic self-powered poly(3-hexylthiophene)/zinc oxide (P3HT/ZnO) heterojunction photodetectors incorporating silver triangular nanoplates (AgTNPs) were fabricated using sol–gel and spin-coating techniques. The experimental results demonstrate that the incorporation of AgTNP nanostructures significantly enhances the photoelectric conversion efficiency of the plasmonic P3HT/AgTNPs/ZnO photodetectors across both the ultraviolet and visible spectral regions. The responsivity enhancement ratio of the plasmonic devices reached its maximum under illumination at a wavelength of 525 nm. Compared with the reference P3HT/ZnO device, the responsivity values of the P3HT/AgTNPs-1/ZnO and P3HT/AgTNPs-2/ZnO devices increased by factors of 3.24 and 4.21, respectively. The optimal P3HT/AgTNPs-2/ZnO device exhibited responsivity values of 9.49, 10.80, and 10.47 mA/W under irradiation at wavelengths of 440 nm, 460 nm, and 525 nm, respectively. The mechanism of performance enhancement induced by the plasmonic AgTNPs is also discussed. This work demonstrates that embedding triangular plasmonic metal nanoplates within semiconductor heterojunctions constitutes an effective strategy for performance enhancement, providing new insights for the rational design of high-performance optoelectronic devices.

## 1. Introduction

The rapid advancement of the Internet of Things (IoT) has spurred increasing demands for high-performance, fast-response sensors; however, sustainable power supply remains a critical bottleneck hindering their widespread deployment [1,2]. In this context, self-powered photodetectors exhibit considerable promise. Capable of autonomous operation without external power supplies, these devices are especially well suited for applications in remote or hazardous environments—such as missile exhaust detection, environmental monitoring, and flame sensing—thereby providing a viable pathway toward fully self-sufficient IoT sensors [3,4,5,6]. Among various semiconductor materials, zinc oxide (ZnO) has garnered significant attention for constructing high-performance self-powered photodetectors due to its inherent wide bandgap (~3.37 eV) and high electron mobility [7]. Furthermore, poly(3-hexylthiophene) (P3HT)—a stable p-type organic semiconductor—exhibits high hole mobility due to its excellent crystallinity and superior charge-transport properties [8,9,10,11]. Consequently, ZnO/P3HT heterojunctions have been widely adopted in photodetectors due to their outstanding optoelectronic performance [12,13].

In the pursuit of high-performance self-powered photodetectors, plasmonic metal nanostructures have been widely used to enhance device efficiency through the local surface plasmon resonance (LSPR) effect. This mechanism significantly enhances the light capture capability. Silver [14,15,16,17] and gold [18,19,20] are the most commonly used plasmonic materials in such field enhancement applications. Compared to gold, Ag has a lower imaginary part of the dielectric function in the visible light region [21,22], and its work function (~4.2 eV) is conducive to the separation of self-powered carriers [23]. Therefore, this study adopted silver as the plasmonic component.

In addition to the selection of the metal, the morphology of the nanoparticles also plays a crucial role in LSPR performance. Geometries with high curvature vertices and edges generate much stronger electromagnetic “hot spots” than isotropic spheres. For example, Lee et al. [24] integrated gold nanostars into photodetectors, achieving a response rate of 5.90 × 10^4^ A/W and a hundredfold enhancement in detectivity. Gu et al. [25] embedded gold triangular nanoantennas into perovskite photodetectors, obtaining a stronger response rate (51 mA/W) and an external quantum efficiency of 12.6%. Zhang et al. [26] fabricated triangular Al/Al_2_O_3_ core–shell structure arrays on 4H-SiC, achieving a response rate of 2.14 A/W. Huang et al. [27,28] demonstrated that triangular silver nanosheets have higher light absorption properties, enabling a current efficiency of 1.83 cd/A and a 200% enhancement in electroluminescence intensity in polymer light-emitting diodes. Although previous studies have confirmed that the geometric shape of metal nanostructures can regulate local surface plasmon resonance through lightning rod effects, thereby improving light absorption and carrier extraction efficiency, current exploration still focuses on configurations such as silver nanospheres, nanorods, and dodecahedrons, with limited research on silver triangular nanoplates. Silver triangular nanoplates not only generate strong local field enhancement with sharp apexes, but they also exhibit high synthesis yields and avoid the strict requirements for dispersion stability associated with complex shapes. Therefore, exploring the plasmon enhancement effect of silver triangular nanoplates is a key challenge for advancing the development of a new generation of high-performance ZnO-based photodetectors.

Based on the above considerations, this study proposes and fabricates a self-powered P3HT/ZnO heterojunction photodetector integrating AgTNPs as an efficient LSPR source. By regulating the loading amount of AgTNPs to achieve precise LSPR enhancement, the responsivity and detectivity of the device are significantly improved. This work fills the research gap of triangular silver nanostructures in ZnO-based devices, clarifies the mechanism of their morphological advantages for photovoltaic performance, and provides clear design guidelines for next-generation self-powered photodetectors for Internet of Things applications.

## 2. Materials and Methods

### 2.1. Materials

Zinc acetate dihydrate (Zn(CH_3_COO)2.2H_2_O, 99%), silver nitrate (AgNO_3_, 99.7%), and acetone (C_3_H_6_O, 99%) were purchased from Sinopharm Chemical Reagent Co., Ltd. (Shanghai, China) Trisodium citrate dihydrate (Na_3_C_6_H_5_O_7_·2H_2_O, 99%), poly (vinyl pyrrolidone) (PVP-K30, Mw~58000), hydrogen peroxide (H_2_O_2_, 30 wt%), sodium borohydride (NaBH_4_, 98%), monoethanolamine (MEA) (C_2_H_7_NO, 99%), 2-methoxyethanol (C_3_H_8_O_2_, 99.5%), chlorobenzene (C_6_H_5_Cl, 99.5%) and isopropanol (C_3_H_8_O, 99%) were sourced from Shanghai Aladdin Biochemical Technology Co., Ltd. Poly(3-hexylthiophene) (P3HT, C_10_H_14_R_2_S, 98%), silver powder (99.9%), and fluorine-doped tin oxide (FTO)-coated glass substrates with a sheet resistance of roughly 30 Ω/cm^2^ were supplied by Advanced Election Technology Co., Ltd. (Shenzhen, China). All chemical reagents and solvents utilized in this study were of analytical grade and employed as received, without undergoing further purification. Deionized water was provided by the laboratory.

### 2.2. Synthesis of AgTNPs

The protocol for synthesizing a AgTNP colloidal solution was developed by modifying previously published procedures [29]. In a typical experiment, a freshly prepared 100 μL of a 0.1 M silver nitrate aqueous solution was gradually added into 50 mL of deionized water under vigorous stirring. Subsequently, 3 mL of a 0.03 M trisodium citrate aqueous solution, 3 mL of a 0.05 M poly(vinylpyrrolidone) aqueous solution, and 120 μL of a 30% hydrogen peroxide solution were sequentially added to the silver nitrate aqueous solution. The mixture was mixed at room temperature with magnetic stirring for 10 min. Then, a freshly prepared 300 μL of a 0.01 M sodium borohydride aqueous solution was rapidly injected into the mixture solution. After 3–5 s, the color of the mixture solution changed from transparent to bright yellow. Subsequently, the color gradually changed from yellow to pink, red, then to purple, and finally to blue. The mixture solution was stirred constantly for another 30 min to achieve equilibrium in the reduction reaction. Then, a blue stable AgTNP colloidal solution was obtained. The blue solution was centrifuged at 10,000 rpm for 20 min. The transparent supernatant was discarded, and the precipitate was retained and washed three times with deionized water. The obtained AgTNP sediment was redispersed in deionized water for subsequent application. All the experiments were carried out at atmospheric pressure.

### 2.3. Device Fabrication

**Figure 1:**
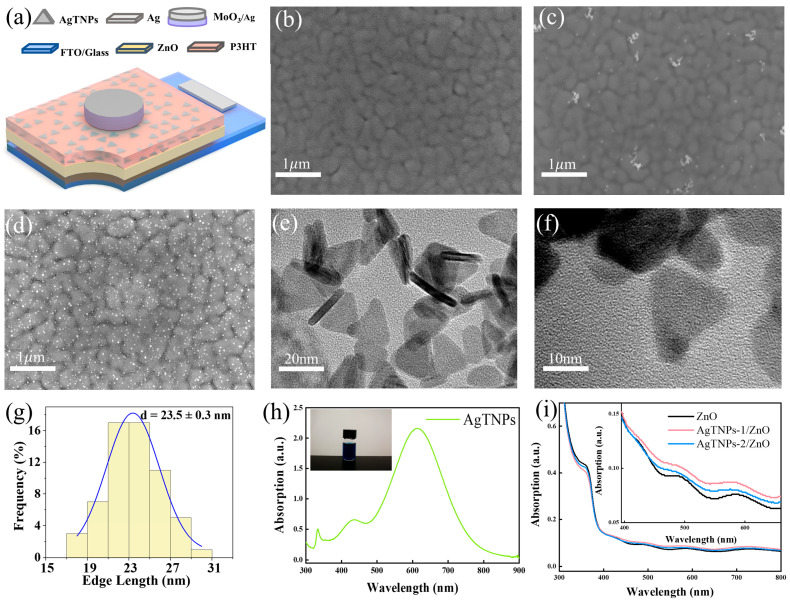
(**a**) Schematic diagram of the P3HT/AgTNPs/ZnO photodetector. (**b**) Top-view FESEM image of the ZnO film. (**c**) Top-view FESEM image of the AgTNPs-1/ZnO film. (**d**) Top-view FESEM image of the AgTNPs-2/ZnO film. (**e**) TEM and (**f**) high-resolution TEM images of colloidal AgTNPs. (**g**) Histogram of AgTNP lengths. (**h**) UV–vis–NIR spectrum of the colloidal AgTNP solution. (**i**) UV–vis–NIR absorption spectra of the ZnO, AgTNPs-1/ZnO, and AgTNPs-2/ZnO films. The inset is the magnified view of the 360–660 nm wavelength region.

The fabrication procedures for the P3HT/ZnO heterojunction photodetectors incorporated with AgTNPs were similar to those in our previous report [30]. Firstly, the FTO substrates were subjected to sequential ultrasonic cleaning in acetone, isopropanol, and deionized water for 10 min in each solvent, followed by drying under a nitrogen stream. Secondly, a ZnO precursor solution was prepared by dissolving zinc acetate (0.3 M) and monoethanolamine (MEA, 0.3 M) in 40 mL of 2-methoxyethanol. This solution was spin-cast onto the cleaned FTO substrates at 2000 rpm for 30 s and subsequently heat-treated at 240 °C for 10 min. The deposition of a ZnO layer was carried out six times in total, with an intermediate annealing step at 385 °C for 10 min performed after each individual coating cycle. Thirdly, the synthesized AgTNP colloidal suspension was applied onto the ZnO film by spin coating at 1200 rpm for 20 s and annealed at 150 °C for 5 min. This procedure was repeated either once or twice. Subsequently, an organic polymer film composed of P3HT (15 mg/mL in chlorobenzene) was deposited over the AgTNP-decorated ZnO layers via spin coating at 3000 rpm for 30 s, and the sample was heated on a hotplate at 150 °C for 30 min. Finally, a 3 nm MoO_3_ layer followed by a 140 nm Ag electrode were thermally evaporated onto the P3HT surface to establish ohmic contact, while a minute quantity of silver paste was placed on the exposed FTO region and allowed to dry for subsequent electrical and optical characterization. At this stage, the AgTNP-modified P3HT/ZnO photodetectors were obtained, referred to as P3HT/AgTNPs-1/ZnO (or J2) device and P3HT/AgTNPs-2/ZnO (or J3) devices when the spin-coating of the AgTNP colloidal solution was executed once and twice, respectively. The schematic diagram of the P3HT/AgTNPs/ZnO device is shown in Figure 1a. For comparison purposes, a P3HT/ZnO hybrid heterojunction photodetector without AgTNPs (designated as J1) was fabricated under identical conditions. The active area of all the photodetectors without or with metal nanoparticles was 0.08 cm^2^.

### 2.4. Characterization

Surface morphologies were investigated via field emission scanning electron microscopy (FESEM, Zeiss SUPRA55VP). TEM (JEOL JEM-2100) was employed to resolve the structural dimensions and shapes of the synthesized AgTNPs. Optical properties were recorded using a Shimadzu UV-2600 UV–vis–NIR spectrophotometer (Shimadzu, Kyoto, Japan). The current versus voltage (I–V) and the time-dependent photocurrent (I-T) characteristics of these as-fabricated devices were analyzed using Keithley 2400 series source meters in the dark or under illumination from light-emitting diode lights (LEDs) at wavelengths of 360 nm, 380 nm, 400 nm, 440 nm, 460 nm, 525 nm, and 660 nm.

## 3. Results and Discussion

Figure 1b–d shows top-view FESEM images of the control ZnO film, the AgTNPs-1/ZnO film, and the AgTNPs-2/ZnO film deposited on FTO/glass substrates, respectively. The images reveal that the films of the two AgTNP-covered ZnO samples exhibit a markedly denser distribution of bright (white) features compared to those with only one layer, indicating a substantial increase in the surface coverage and total deposition amount of AgTNPs. Figure 1e,f show TEM images of the colloidal AgTNPs, unambiguously revealing their near-equilateral triangular morphology. This shape results from preferential crystal growth along the (100) or (110) lattice planes, which is directed by surfactant-mediated facet stabilization [31,32]. Figure 1g displays the length histogram of the AgTNPs, showing a nanoplate edge-length distribution ranging from 15 to 31 nm, which is well approximated by a normal distribution. Figure 1h presents the UV–vis–NIR absorption spectrum of the colloidal AgTNP solution over the wavelength range of 300–900 nm. A distinct absorption peak was observed at approximately 615 nm, corresponding to the dipole mode of the AgNPs [33]. This peak is slightly red-shifted compared to the dipole-mode LSPR typically exhibited by spherical silver nanoparticles. This red shift is attributed to the increased edge length of the triangular nanosheets, which enhances charge separation; the localization of electrons at the tips, which increases the spacing between positive ions; and the higher refractive index of the device dielectric compared to that of the sol-state [34]. Figure 1i compares the UV–vis–NIR spectra of the ZnO, AgTNPs-1/ZnO, and AgTNPs-2/ZnO films on FTO/glass substrates. The absorption below 310 nm originates primarily from the FTO/glass substrate. Owing to the characteristic band-edge absorption of the ZnO layer, all three devices exhibit strong ultraviolet absorption below 360 nm [35]. The inset provides an expanded view of the 360–660 nm wavelength region. It illustrates that the absorption of the samples with AgTNPs is slightly higher in the 400–660 nm range [32,36,37].

**Figure 2:**
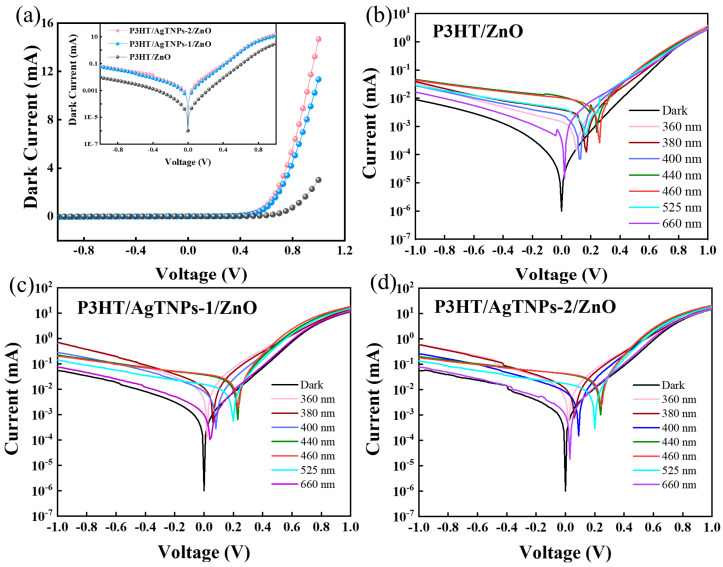
(**a**) *I*–*V* characteristic curves of the as-fabricated J1, J2, and J3 devices under dark conditions in linear scale and semilogarithmic scale (inset). *I*–*V* characteristic curves of J1 (**b**), J2 (**c**), and J3 (**d**) devices under dark conditions and illumination under LED light with wavelengths of 360 nm, 380 nm, 400 nm, 440 nm, 460 nm, 525 nm, and 660 nm.

Figure 2a presents the I–V characteristic curves of the P3HT/ZnO heterojunction photodetectors with and without AgTNPs under dark conditions over a bias voltage range of −1 V to +1 V. The figure shows that the I-V characteristics of all three heterojunction photodetectors exhibit typical diode-like rectifying behaviors. This excellent rectification capability arises from the precise band energy level alignment across the Ag/MoO_3_/P3HT/ZnO/Ag multilayer structure. To systematically evaluate and compare the self-powered performance of devices J1, J2, and J3, I–V measurements were conducted both in the dark and under illumination by monochromatic LEDs at wavelengths of 360, 380, 400, 440, 460, 525, and 660 nm. The corresponding I–V curves are shown in Figure 2b–d. These results demonstrate that all three devices generate pronounced photovoltaic responses across the ultraviolet-to-visible spectral range. Notably, under identical experimental conditions, J2 and J3 yield significantly higher photocurrents than J1.

**Figure 3:**
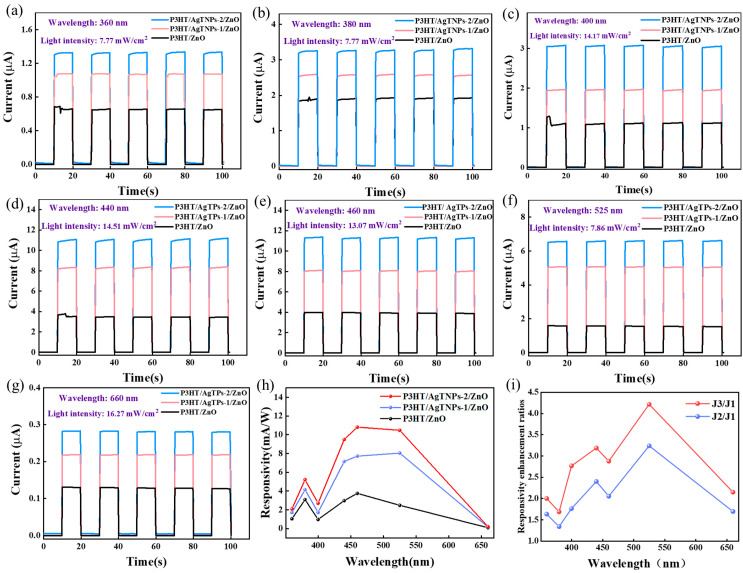
*I-T* curves of the J1, J2, and J3 devices illuminated by LED with wavelengths of (**a**) 380 nm (7.77 mW/cm^2^), (**b**) 380 nm (7.77 mW/cm^2^), (**c**) 400 nm (14.17 mW/cm^2^), (**d**) 440 nm (14.51 mW/cm^2^), (**e**) 460 nm (13.07 mW/cm^2^), (**f**) 525 nm (7.86 mW/cm^2^), and (**g**) 660 nm (16.27 mW/cm^2^). (**h**) The calculated responsivity values of the J1, J2, and J3 photodetectors as a function of illumination light wavelength. (**i**) Responsivity enhancement ratio of J2, and J3 compared to J1 device as a function of illumination light wavelength.

To investigate the optical response performance of self-powered P3HT/ZnO heterojunction photodetectors modulated by the LSPR effect of AgTNPs, the temporal response characteristics of devices J1, J2, and J3 were measured under zero-bias conditions using LED illumination at wavelengths of 360 nm, 380 nm, 400 nm, 440 nm, 460 nm, 525 nm, and 660 nm. Specifically, illumination was cycled on and off every 20 s for a total of five complete cycles. Figure 3a–g show the time-resolved photoresponse characteristics of the three devices under zero bias, measured at wavelengths of 360 nm, 380 nm, 400 nm, 440 nm, 460 nm, 525 nm, and 660 nm, respectively. The experimental results demonstrate that all devices exhibit a rapid current increase upon illumination onset and a similarly rapid decay upon illumination termination—highlighting excellent photoresponse repeatability and robust switching performance [38]. Without irradiation, all devices exhibit a low dark current (I_dark_). Upon illumination, the current rapidly rises and stabilizes at the corresponding photocurrent plateau (I_light_). Specifically, it is observed that the J2 and J3 devices with AgTNPs exhibit higher photocurrent values compared with the J1 device under the same light irradiation conditions. The photocurrent values of the J1, J2, and J3 devices are 3.92 μA, 8.06 μA, and 11.30 μA, respectively, under monochromatic illumination from a 460 nm LED with an irradiance intensity of 13.07 mW/cm^2^. These results demonstrate that strategic integration of AgTNPs into the ZnO/P3HT self-powered photodetectors can effectively increase the photocurrent of the devices. The key metrics for evaluating photodiode performance include responsivity (R), photodetector current ratio (PDCR), noise equivalent power (NEP), normalized detection efficiency (D*), linear dynamic range (LDR), and external quantum efficiency (EQE). These parameters can be expressed by the formulas below [39,40,41,42].

(1) $$R=\frac{I_{photo}-I_{dark}}{AP}$$

(2) $$PDCR=\frac{I_{photo}}{I_{dark}}$$

(3) $$NEP=\frac{I_{noise}}{R}=\frac{\sqrt{2q\times I_{dark}}}{R}$$

(4) $$D*=\frac{R}{\sqrt{2q\times \frac{I_{dark}}{S}}}$$

(5) $$LDR=20log(\frac{I_{photo}}{I_{dark}})$$

(6) $$EQE=R\times \frac{hc}{q\lambda }\times 100\%$$

where A is the active area (0.08 cm^2^), h is Planck’s constant, c is the speed of light, q is the elementary charge, and λ is the wavelength of the irradiation light. The calculated values of R, D*, EQE, PDCR, NEP, and LDR for the J1, J2 and J3 devices based on the above formulas are summarized in Table 1. The calculated R values curves and R enhancement ratio values as a function of illumination light wavelength are plotted in Figure 3h and Figure 3i, respectively. It can be observed that the J2 and J3 devices have higher R values compared with the J1 device under the same light irradiation conditions. In particular, for the J3 device, when excited by light at wavelengths at 440 nm, 460 nm and 525 nm, the R values were 9.49, 10.80 and 10.47 mA/W. The R enhancement ratio values in Figure 3i show that the devices modified with AgTNPs exhibit the best enhancement effect on the photoresponsivity under irradiation at 525 nm. Compared with the J1 device, the R values of the J2 and J3 devices increased by factors of 3.24 and 4.21 times, respectively. Notably, the peak device enhancement at 525 nm precisely coincides with the intrinsic absorption maximum of the P3HT active layer, rather than the localized surface plasmon resonance (LSPR) wavelength of the AgTNPs observed in thin films (615 nm, Figure 1h). This discrepancy indicates that the primary enhancement mechanism is the near-field enhancement and scattering effect of the AgTNPs acting as optical antennas, rather than hot-electron injection. However, the device response is governed by the spectral overlap with the active layer’s absorption. Under resonant illumination, a strongly amplified local electromagnetic field arises around the AgTNPs. This field enhancement boosts the optical absorption of the neighboring P3HT and ZnO photosensitive layers, ultimately elevating the photon-to-electron conversion efficiency of the self-powered P3HT/ZnO heterojunction photodetector [29,31]. At wavelengths far from the enhanced absorption window (e.g., 360 nm, 380 nm, and 660 nm), the field enhancement effect weakens, resulting in a relatively smaller increase in responsivity. Concurrently, the pronounced light-scattering capability of the AgTNP architecture effectively elongates the optical path length within the active layer [32,35], thereby enhancing photon capture and absorption efficiency in both the ZnO and P3HT layers [36]. Furthermore, hot electrons generated at the metal–semiconductor interface under resonant excitation may surmount the Schottky barrier and be injected into the conduction band of ZnO as a secondary contribution. The built-in electric field arising from the metal–semiconductor junction promotes rapid separation and efficient extraction of photogenerated carriers while simultaneously suppressing electron–hole recombination [32]. Owing to the synergistic enhancement effects of AgTNPs—encompassing localized near-field confinement, optical path elongation, and facilitated charge separation—both the light absorption and photon-to-electron conversion efficiency of the P3HT/AgTNPs-2/ZnO heterojunction photodetector are substantially improved, culminating in a marked enhancement of its optical responsivity.

The calculated values of D*, EQE, PDCR, NEP, and LDR for the J1, J2, and J3 devices are summarized in Table 1. As shown in Table 1, the trend in EQE values across the J1, J2, and J3 devices is largely consistent with that of the responsivity (R) values. Notably, for the J3 device under illumination at 440 nm, 460 nm, and 525 nm, the corresponding EQE values are 2.67%, 2.91%, and 2.47%, respectively. Table 1 further reveals that although incorporation of AgTNPs enhances photon utilization efficiency, it concurrently degrades dark-state performance and compromises detection sensitivity. Specifically, the dark current increases in both the J2 and J3 devices containing AgTNPs, as evidenced by the inset in Figure 2a. This elevated dark current results in a reduced PDCR, a lower specific D*, a higher NEP, and a narrower LDR—all indicative of an increased minimum detectable optical power and, consequently, diminished capability for low-light detection in practical applications. This observation highlights a fundamental trade-off intrinsic to plasmonic enhancement: while metallic nanostructures improve light absorption, they simultaneously introduce additional leakage pathways and non-radiative recombination centers.

Beyond the internal comparison among J1–J3, we further benchmarked the optimized P3HT/AgTNPs-1/ZnO device against representative self-powered ZnO-based photodetectors reported in the literature (Table 2). Under zero-bias operation and 460 nm illumination (10 mW/cm^2^), our device achieves a specific detectivity of 8.25 × 10^10^ Jones and an EQE of 2.91%. This detectivity exceeds those of P3HT/ZnO (2.74 × 10^7^ Jones), p^+^-FLG/n^−^-ZnO NW (1.9 × 10^9^ Jones), and ZnONRs/Au/PEDOS (1.16 × 10^10^ Jones), and is comparable to that of Au NPs@ZnO NWs (2.75 × 10^11^ Jones) despite the latter being measured at a much lower light intensity. Moreover, the EQE of 2.91% surpasses the value reported for pristine P3HT/ZnO (1.05%). The enhanced performance can be attributed to the incorporation of Ag triangle nanoplates, which facilitate localized surface plasmon resonance and improved charge separation at the P3HT/ZnO interface. Overall, this external benchmarking confirms that the P3HT/AgTNPs/ZnO heterostructure offers a favorable combination of detectivity and visible-light conversion efficiency among self-powered ZnO-based detectors.

**Figure 4:**
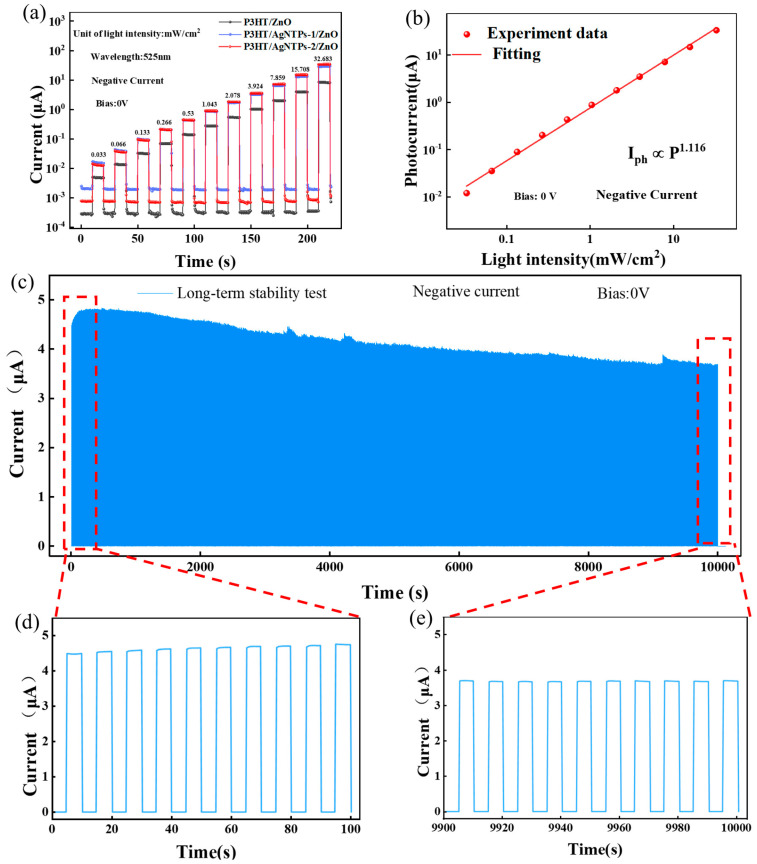
(**a**) Semilogarithmic scale *I-T* curves of the J3 (P3HT/AgTNPs-2/ZnO) device under 525 nm light irradiation with different excitation intensities ranging from 0.033 to 32.683 mW/cm^2^ at zero-bias voltage. (**b**) Photocurrent as a function of light intensity and the corresponding power law fitting curves. (**c**) Dynamic response of J3 (P3HT/AgTNPs-2/ZnO) under on/off modulated light in a test period of 1000 cycles under 525 nm light. (**d**) Magnified details of photocurrent during the first 10 cycles. (**e**) Magnified details of photocurrent during the last 10 cycles.

Figure 4a shows the semi-logarithmic I-T curves of the fabricated P3HT/AgTNPs-2/ZnO heterojunction photodetector under zero bias and light intensities ranging from 0.033 to 32.683 mW/cm^2^. With increasing incident light power density, the photocurrent of all photodetectors exhibits a continuous upward trend. To investigate the photoresponse characteristics of the device, we examined the dependence of photocurrent on excitation light intensity. The relationship between photocurrent and excitation light intensity is depicted in Figure 4b and can be fitted by the equation I_ph_ ∝ P_q_, where P represents incident light intensity and q denotes the power-law exponent. Fitting the curve over a broad intensity range yielded a q value of 1.116 for this device. This result also reflects the device’s excellent photosensitivity, with high stability in both photogenerated carrier generation and collection efficiency across the tested light intensity range.

In practical applications, operational stability and storage stability are of paramount importance. To systematically evaluate device performance, the optimized P3HT/AgTNPs-2/ZnO photodetector was subjected to 1000 consecutive switching cycles under zero-bias conditions and monochromatic illumination at 525 nm. The corresponding current–time (I–T) response curve is shown in Figure 4a, while expanded views of the initial and final cycles are presented in Figure 4d and Figure 4e, respectively. Quantitative analysis indicates that the photocurrent retains more than 82% of its initial value after 1000 cycles, demonstrating exceptional operational durability. The slight performance degradation is likely attributable to intrinsic photoinduced fatigue effects in the active layer material under prolonged illumination. This outstanding operational reliability and stability strongly support the detector’s promising potential for broad applications in optical sensing and imaging technologies.

## 4. Conclusions

In this study, plasmonic self-powered P3HT/ZnO heterojunction photodetectors modified with AgTNPs were successfully fabricated. By precisely embedding AgTNPs—engineered with well-defined geometric shapes—into the interface between the ZnO and P3HT layers, the interfacial properties and light-trapping capability of the heterojunction were effectively modulated. The experimental results demonstrate that incorporating AgTNPs into the device significantly enhances the photoelectric conversion efficiency across both the ultraviolet and visible spectral regions. For the optimized P3HT/AgTNPs-2/ZnO device, the R reaches 9.49, 10.80, and 10.47 mA/W under illumination at 440 nm, 460 nm, and 525 nm, respectively, corresponding to EQE values of 2.67%, 2.91%, and 2.47%. The maximum R enhancement is observed at 525 nm: the responsivities of the P3HT/AgTNPs-1/ZnO and P3HT/AgTNPs-2/ZnO devices increase by 3.24-fold and 4.21-fold, respectively, relative to the reference P3HT/ZnO device. This pronounced improvement is primarily ascribed to the LSPR effect uniquely exhibited by AgTNPs. Moreover, the device retains excellent operational stability after 1000 consecutive photocurrent measurement cycles, underscoring its robust reliability. This work not only highlights the considerable potential of AgTNPs in boosting optoelectronic performance but also offers a rational materials design strategy for developing next-generation low-cost, high-efficiency, self-powered photodetectors.

## 5. Prospects

Although this study has demonstrated the significant advantages of AgTNPs in improving the response performance of P3HT/ZnO heterojunction photodetectors, there are still several issues that need to be further clarified and resolved in future work. Further research should be pursued in four directions: Firstly, FDTD or COMSOL Multiphysics 6.2 optical simulations are needed to quantify the local field distribution at triangular tips and its correlation with LSPR enhancement. Secondly, surface passivation or dielectric isolation layers should be explored to suppress the dark current and instability originating from highly active tips. Thirdly, a statistically robust quantitative analysis of AgTNP loading and surface coverage from SEM images is required to establish a definitive structure–performance relationship. Fourthly, long-term storage and environmental stability tests must be conducted to assess feasibility for practical IoT deployment. These investigations will collectively deepen the understanding of the plasmon-enhancement mechanism of AgTNPs and guide the design of self-powered ZnO-based photodetectors for IoT applications.

## Figures and Tables

**Table 1:** The calculated *R*, D*, *EQE*, *PDCR*, *NEP*, and *LDR* values of J1, J2 and J3 devices illuminated with light at wavelengths of 360 nm, 380 nm, 380 nm, 400 nm, 440 nm, 525 nm and 660 nm.

Device	Wavelength (nm)	R (mA/W)	D* (Jones)	EQE (%)	PDCR	NEP (W)	LDR (dB)
J1	360	1.05	7.25 × 10^10^	0.36	12,451	3.90 × 10^−12^	81.90
	380	3.09	1.65 × 10^11^	1.0	22,153	1.70 × 10^−12^	86.90
	400	0.97	1.05 × 10^11^	0.30	51,336	2.69 × 10^−12^	94.205
	440	2.98	2.17 × 10^11^	0.84	73,727	1.30 × 10^−12^	97.35
	460	3.75	1.92 × 10^11^	1.01	41,310	1.46 × 10^−12^	92.321
	525	2.48	1.13 × 10^11^	0.59	12,985	2.49 × 10^−12^	82.26
	660	0.099	3.99 × 10^9^	0.02	842	7.08 × 10^−11^	58.51
J2	360	1.72	1.87 × 10^10^	0.59	511	1.50 × 10^−11^	54.17
	380	4.14	4.65 × 10^10^	1.35	1299	6.07 × 10^−12^	62.27
	400	1.72	2.00 × 10^10^	0.53	1062	1.40 × 10^−11^	60.52
	440	7.15	7.12 × 10^10^	2.01	3302	3.96 × 10^−12^	70.37
	460	7.72	1.25 × 10^11^	2.08	8481	2.26 × 10^−12^	78.56
	525	8.04	1.51 × 10^11^	1.90	7183	1.86 × 10^−12^	77.12
	660	0.17	3.39 × 10^9^	0.03	360	8.32 × 10^−11^	51.13
J3	360	2.10	8.54 × 10^9^	0.72	87	3.30 × 10^−11^	38.82
	380	5.21	2.04 × 10^10^	1.70	201	1.38 × 10^−11^	46.07
	400	2.70	1.78 × 10^10^	0.84	536	1.58 × 10^−11^	54.58
	440	9.49	6.14 × 10^10^	2.67	1850	4.60 × 10^−12^	65.34
	460	10.80	8.25 × 10^10^	2.91	2642	3.42 × 10^−12^	68.43
	525	10.47	8.16 × 10^10^	2.47	1601	3.46 × 10^−12^	64.08
	660	0.21	1.51 × 10^9^	0.04	57	1.86 × 10^−10^	35.18

**Table 2:** Comparison of the detection parameters of ZnO photodetectors reported in recent works.

Device	Wavelength (nm)	Light Intensity (mW/cm^2^)	R (mA/W)	D* (Jones)	EQE (%)
GZO [43]	257	0.15	24.4	6 × 10^15^	
P3HT/ZnO [44]	360	10	3.04	2.74 × 10^7^	1.05
Au NPs@ZnO NWs [45]	325	0.068	0.485	27.49 × 10^10^	
p+-FLG/n−-ZnO NW [46]	350	30	120	1.9 × 10^9^	
ZnONRs/Au/PEDOS [47]	365	0.3	0.369	1.16 × 10^10^	
J2	460	10	10.80	8.25 × 10^10^	2.91

## Data Availability

The original contributions presented in this study are included in the article/Supplementary Material. Further inquiries can be directed to the corresponding author.

## Supplementary Materials

The following supporting information can be downloaded at: https://www.mdpi.com/article/10.3390/s26092725/s1.
