# Supplementary figures and images for: High-Performance Self-Powered Photodetector Based on Silver Triangular Nanoplate-Modified P3HT/ZnO Heterojunctions
Source: Sensors (Basel). 2026 Apr 28;26(9):2725. doi: 10.3390/s26092725 (PMC13165943; doi:10.3390/s26092725)

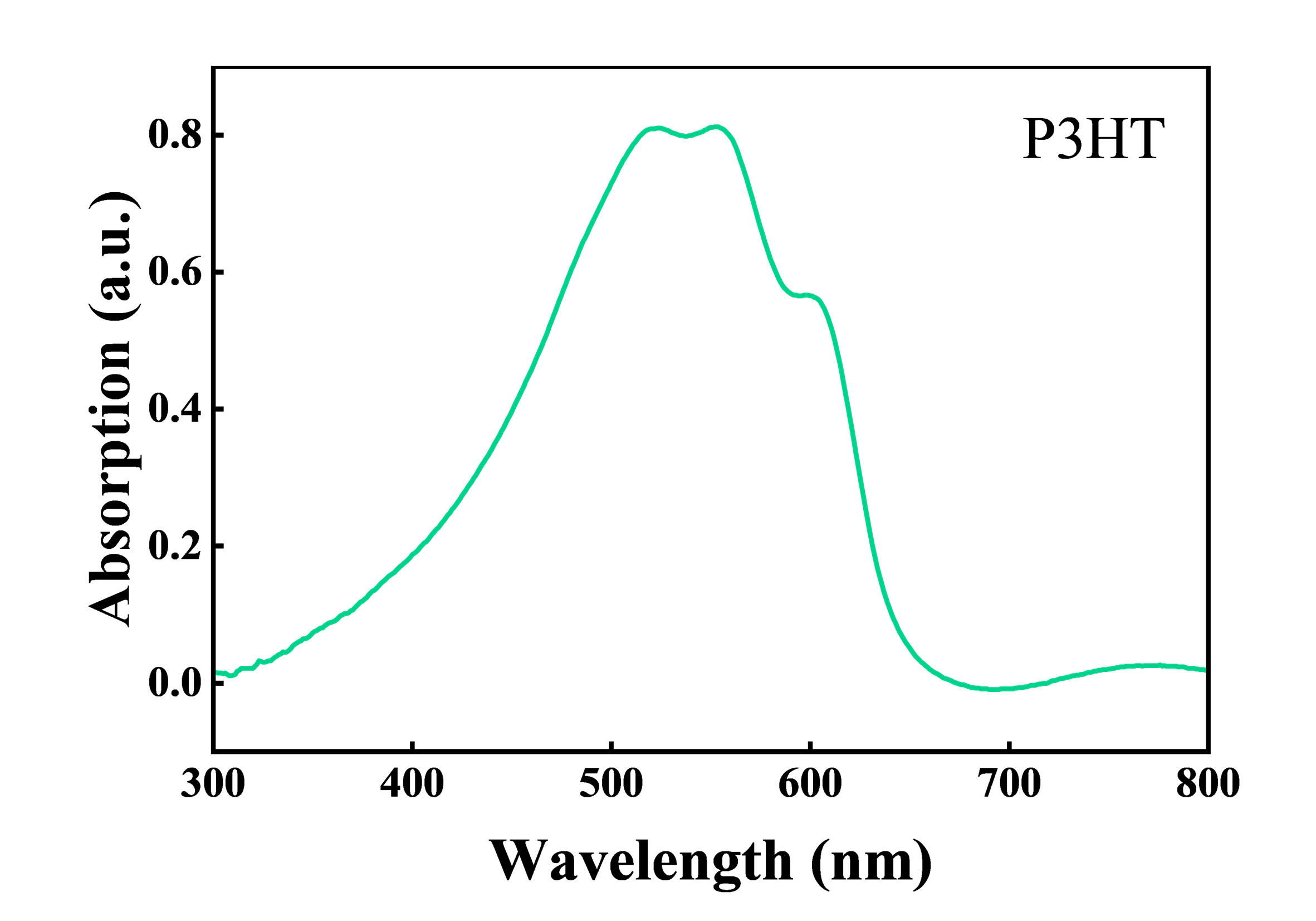

Supplement: Supplementary file 1 [file sensors-26-02725-s001.zip › sensors-4242925-supplementary.tif]
